# Successful closure of a refractory gastrobronchial fistula using endoscopic mucosal ablation followed by single loop-and-clips technique

**DOI:** 10.1055/a-2127-4663

**Published:** 2023-08-21

**Authors:** Sandra Maisterra, Sergi Quintana-Carbo, Humberto Aranda, Mariona Calvo, Leandre Farran, Nuria Virgili, Joan B. Gornals

**Affiliations:** 1 Endoscopy Unit, Department of Digestive Diseases, Hospital Universitari de Bellvitge, Barcelona, Catalonia, Spain; 2 Bellvitge Biomedical Research Institute (IDIBELL), Barcelona, Catalonia, Spain; 3 Universitat de Barcelona, Barcelona, Catalonia, Spain; 4 Gastroesophageal Tumours Functional Unit (UTEG), Hospital Universitari de Bellvitge, Institut Català d’Oncologia, Barcelona, Spain; 5 General and Digestive Surgery Department, Hospital Universitari de Bellvitge, Barcelona, Catalonia, Spain; 6 Medical Oncology Department, Institut Català dʼOncología (ICO), Barcelona, Spain; 7 Endocrinology and Clinical Nutrition department, Hospital Universitari de Bellvitge, Barcelona, Catalonia, Spain

A 78-year-old man developed a late fistula between the gastroplasty pouch and right bronchus following Ivor Lewis esophagectomy for esophageal neoplasia ( Fig. 1 ). He was unable to ingest food orally, necessitating the placement of a permanent percutaneous jejunostomy. He had a persistent cough, and the fistula was refractory to various endoscopic interventions, including placement of clips, tissue adhesives, and stents.

**Fig. 1:**
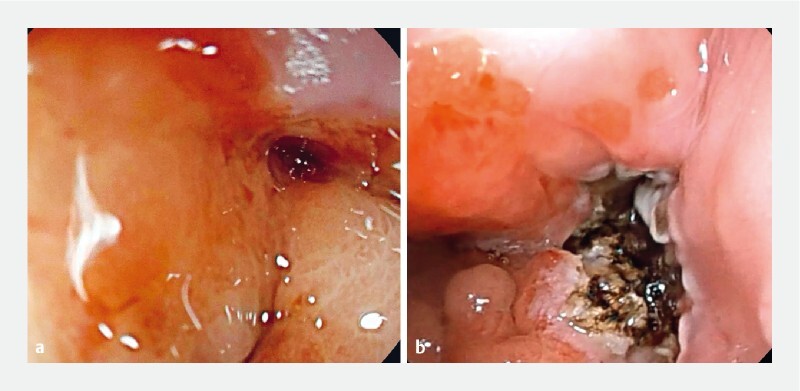
Endoscopic images of the post-esophagectomy fistula **a** before and **b** after endoscopic mucosal ablation using argon plasma coagulation.

In the first session, the fistula was treated using a combination of endoscopic submucosal dissection (ESD) and over-the-scope (OTS) clip. ESD was performed around and inside the fistula tract, aiming to create a 1-cm mucosal patch that was centrally positioned at the orifice ( Video 1 ). To extract the fistula from the wall, traction was applied to the mucosal flap using the clip-with-line traction technique, enabling deeper dissection of the fistula tract. Finally, an OTS clip was deployed to close the orifice. However, after a period of 7 days, the patient experienced a recurrence of symptoms due to the detachment of the OTS clip from the gastrointestinal wall.

**Video 1** Ultimately successful closure of a refractory gastrobronchial fistula using the combination of endoscopic submucosal dissection (ESD) and application of an over-the-scope (OTS) clip, followed later by argon ablation and single loop-and-clip technique.


During the second session, a persistent 5-mm fibrotic fistula orifice was observed. To address this, a combination of mucosal ablation and the single loop-and-clips technique (“King” closure) was performed ( Fig. 2 ). Endoscopic mucosal ablation was performed using argon plasma coagulation targeting the intrafistular mucosa and a surrounding circumferential area to eliminate the mucosal scar tissue and promote the healing of the fistular orifice ( Fig. 1 ). Next, the King closure technique was successfully applied using five clips and a coaxial polyloop. Immediate post-procedure capnography did not detect the presence of CO _2_ . The follow-up at 8 weeks after the procedure confirmed fistula resolution based on the absence of symptoms and no contrast leakage on imaging ( Fig. 3 ). At the 1-year follow-up, the patient remained asymptomatic.

**Fig. 2 a, b:**
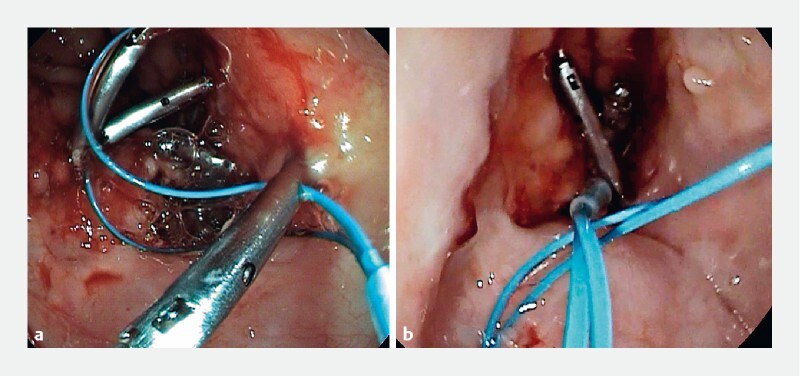
Gastrobronchial fistula closure using the single-loop-and-clips technique.

**Fig. 3:**
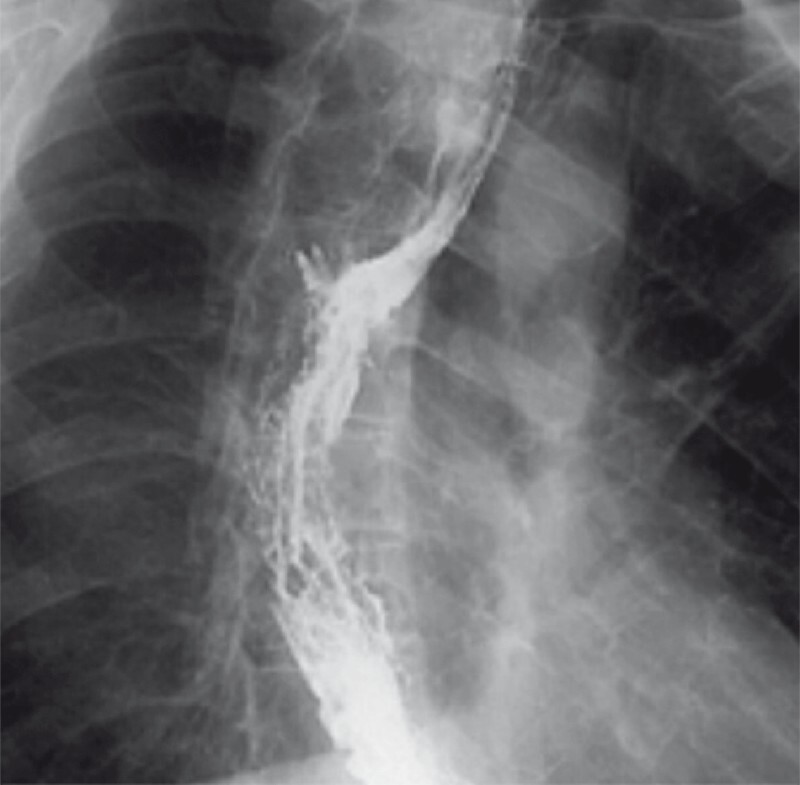
Esophagogram showing no signs of persistence of fistula.

The management of esophagobronchial and gastrobronchial fistulas is challenging. Recently, some French investigators have reported the use of ESD followed by the application of an OTS clip for the treatment of severe fistulas. However, this method can fail in some cases 1 2 3 . Studies have reported that the King closure is a safe technique and provides superior long-term histological healing outcomes compared with OTS clipping 4 5 . In conclusion, perifistular mucosal ablation followed by the single loop-and-clips technique appears to be an effective approach for the treatment of complex fistulas.

Endoscopy_UCTN_Code_CPL_1AH_2AG

**Correction**

**Correction: Successful closure of a refractory gastrobronchial fistula using endoscopic mucosal ablation followed by single loop-and-clips technique** Maisterra S, Quintana-Carbo S, Aranda H et al. Successful closure of a refractory gastrobronchial fistula using endoscopic mucosal ablation followed by single loop-and-clips technique. Endoscopy 2023; 55: E944–E945. In the above-mentioned article, the video has been replaced. This was corrected in the online version on December 19, 2024.

## Notes

Endoscopy E-Videos

https://eref.thieme.de/e-videos
*E-Videos* is an open access online section of the journal *Endoscopy* , reporting on interesting cases and new techniques in gastroenterological endoscopy. All papers include a high-quality video and are published with a Creative Commons CC-BY license. Endoscopy E-Videos qualify for HINARI discounts and waivers and eligibility is automatically checked during the submission process. We grant 100% waivers to articles whose corresponding authors are based in Group A countries and 50% waivers to those who are based in Group B countries as classified by Research4Life (see: https://www.research4life.org/access/eligibility/ ). This section has its own submission website at https://mc.manuscriptcentral.com/e-videos

## Notes

Correction **Correction: Successful closure of a refractory gastrobronchial fistula using endoscopic mucosal ablation followed by single loop-and-clips technique** Maisterra S, Quintana-Carbo S, Aranda H et al. Successful closure of a refractory gastrobronchial fistula using endoscopic mucosal ablation followed by single loop-and-clips technique. Endoscopy 2023; 55: E944–E945. In the above-mentioned article, the video has been replaced. This was corrected in the online version on December 19, 2024.
