# Correction: Successful closure of a refractory gastrobronchial fistula using endoscopic mucosal ablation followed by single loop-and-clips technique

**DOI:** 10.1055/a-2505-9074

**Published:** 2024-12-19

**Authors:** Sandra Maisterra, Sergi Quintana-Carbo, Humberto Aranda, Mariona Calvo, Leandre Farran, Nuria Virgili, Joan B. Gornals

**Affiliations:** 1 Endoscopy Unit, Department of Digestive Diseases, Hospital Universitari de Bellvitge, Barcelona, Catalonia, Spain; 2 Bellvitge Biomedical Research Institute (IDIBELL), Barcelona, Catalonia, Spain; 3 Universitat de Barcelona, Barcelona, Catalonia, Spain; 4 Gastroesophageal Tumours Functional Unit (UTEG), Hospital Universitari de Bellvitge, Institut Català d’Oncologia, Barcelona, Spain; 5 General and Digestive Surgery Department, Hospital Universitari de Bellvitge, Barcelona, Catalonia, Spain; 6 Medical Oncology Department, Institut Català dʼOncología (ICO), Barcelona, Spain; 7 Endocrinology and Clinical Nutrition department, Hospital Universitari de Bellvitge, Barcelona, Catalonia, Spain

**Correction**

**Correction: Successful closure of a refractory gastrobronchial fistula using endoscopic mucosal ablation followed by single loop-and-clips technique** Maisterra S, Quintana-Carbo S, Aranda H et al. Successful closure of a refractory gastrobronchial fistula using endoscopic mucosal ablation followed by single loop-and-clips technique. Endoscopy 2023; 55: E944–E945. In the above-mentioned article, the video has been replaced. This was corrected in the online version on December 19, 2024.

